# FAM64A Promotes Osteosarcoma Cell Growth and Metastasis and Is Mediated by miR-493

**DOI:** 10.1155/2020/2518297

**Published:** 2020-02-24

**Authors:** Ying Jiang, Chunlei Zhou, Qiang Gao, Zhi-Qi Yin, Jingwen Wang, Hong Mu, Jun Yan

**Affiliations:** ^1^Department of Clinical Laboratory, Tianjin First Center Hospital, Tianjin 300192, China; ^2^Department of Pathology, Tianjin First Center Hospital, Tianjin 300192, China

## Abstract

Aberrant expression of FAM64A was correlated with cell proliferation in various cancer types. We examined the expression of FAM64A and the upstream gene miR-493 in OS. The functions of miR-493 were revealed through extensive experiments. We found an increase of FAM64A gene and protein in OS tissues. Overexpression of FAM64A resulted in promoting tumor proliferation, migration, and invasion. The miR-493 targeted and negatively regulated FAM64A. Our data showed that upregulation of FAM64A in OS correlated with poor prognosis.

## 1. Introduction

Osteosarcomas are originated from primitive mesenchymal cells and defined as the most prevalent primary solid tumor of the bone. Due to the heterogeneity of osteosarcoma, the etiology of OS in most patients is still obscure. Fletcher et al. determined increased incidence of OS in cases with altered tumor suppressor genes [1]. For example, CATS (FAM64A) is confirmed to be highly expressed in leukemia, lymphoma, and a range of tumor cell lines. Moreover, it has been reported that its protein levels have intense relationship with proliferation in both tumor cells and nonmalignant cells. Silencing of FAM64A resulted in decreased proliferation and cell cycle progression of hematopoietic cells [2]. However, the research of FAM64A is inadequate; thus, the role of FAM64A in OS cells is still poorly understood.

The dysregulation of intracellular signaling pathways such as Notch1, Akt, Wnt pathway, and JAK2/STAT3 was reported to be participated in the development of OS [3–6]. Xu et al. found that FAM64A served as a positive regulator of STAT3, which is linked to various cancer types [7]. Here, we tried to find a new mechanism that interacts with FAM64A in OS. In this study, we provide an insight into the expression patterns of genes in OS and control samples. We identified the genomic aberrations and the molecular mechanism associated with OS. We discovered the tumor-derived miR-493 targeted to FAM64A and regulated the cell growth and metastasis of OS.

## 2. Materials and Methods

### 2.1. Genomic Data of OS

From Gene Expression Omnibus (GEO; available at http://www.ncbi.nlm.nih.gov/geo/) database, we retrieved the gene expression profiling of OS. The keywords including *Homo sapiens* and OS were used. The informatics analysis of FAM64A levels in OS was performed in the Cancer Genome Atlas (TCGA) database.

### 2.2. Clinical Specimens

From May 2016 to May 2019, 30 patients in total including 17 males and 13 females (average age: 29 years, range: 19 to 46 years) diagnosed with spinal osteosarcoma were included in this study at our hospital (China). All the patients in this study have not received any type of treatment before surgery. Tumor tissues and adjacent normal tissues from patients were stored at −80°C. The protocol for this study was approved by the Ethics Committee at our local institution.

### 2.3. Cell Lines

Human osteosarcoma MG63, U-20S cells, and 293T cells were purchased from FuHeng Cell Center, Shanghai, China. Cells were cultured in DMEM (Dulbecco's modified Eagle's medium, Gibco, Life Technologies) containing 10% (v/v) fetal bovine serum (Gibco, Life Technologies) at 37°C in a humidity incubator with 5% CO_2_.

### 2.4. Xenograft Model of Tumor

For the xenograft tumor model, 1 × 10^7^ U937 MG-63-Scramble/MG-63-siFAM64A transfected cells were implanted into the 12-week-old C57BL/6 mice. Tumor was measured after tumor cells were injected for 2 weeks.

### 2.5. Cell Transfection

The plasmid used was pCDH-CMV-MCS-EF1-Puro. The miR-493 mimics and negative control (miR-NC) were purchased from Shanghai GenePharma (China). After the tumor cells were grown into 75% confluence in 6-well plates, miR-493 mimics (100 pmol) and miR-NC (100 pmol) were used for transfection with the help of Lipofectamine 2000 (Invitrogen; Thermo Fisher Scientific).

### 2.6. Cell Viability Assay

OS cell viability was measured with A CCK-8 assay (Dojindo Molecular Technologies, Japan). Specifically, cells with a density of 7,000 cells/well were firstly seeded in 96-well plates. After 6 h of culture, cells were transfected with MG-63-NC, MG-63-FAM64, U-2 OS-NC, and U-2 OS-FAM64A, respectively, and then incubated at 37°C with 5% CO_2_ for 0, 24, 48, and 72 h, respectively. At each time-point, a CCK-8 reagent of 10 *μ*l was added into each well, and the incubation was subsequently extended for an additional 2 h at 37°C. The absorbance of each well was measured with a microreader (Bio-Rad, USA) at 450 nm.

### 2.7. Cell Invasion Assay

The invasiveness of OS cells was measured with a Transwell assay using Transwell chambers (8 *μ*m, Corning) preburden Matrigel (BD, USA). In detail, cells were first collected and then resuspended in the FBS-free culture medium at a density of 2 × 10^5^ cells/ml. After that, the upper chambers were then seeded with 200 *μ*l cell suspension, while the lower chambers were filled with 600 *μ*l DMEM containing 10% FBS. Following a further incubation for 48 h at 37°C, the noninvasive cells were removed by cotton-tipped swabs. Images of invasive cells were taken, and the number was counted under Nikon ECLIPSE TS100 (Nikon) at ×100 magnification.

### 2.8. Dual‐Luciferase Activity Assay

PCR was used to amply the 3′UTR of FAM64A containing the potential miR-493 binding site. 293T cells were then cotransfected with NC/494 mimics and psiCHECK2-FAM64A3-UTR WT/psiCHECK2-FAM64A3-UTR MT. The dual-luciferase reporter assay (Promega) was then used to measure the relative luciferase activity.

### 2.9. Q-RTPCR

The total RNA from tissues and cells was isolated with TRIzol (Thermo Fisher Scientific, Inc.). In terms of miR-493, U6 was used as an internal reference. Following the synthesis of cDNA using a TaqMan MicroRNA Reverse Transcription kit (Applied Biosystems; Thermo Fisher Scientific, Inc.), PCR was then carried out with the TaqMan MicroRNA PCR kit (Applied Biosystems; Thermo Fisher Scientific, Inc.). The primers used in this study were as follows: miR-493 forward, 5′-TTGTACATGGTAGGCTTTCATT-3′ and reverse 5′-AACCATTTATTTCTCCCGACC-3; U6 forward, 5′-GCTTCGGCAGCACATATACTAAAAT-3′ and reverse 5′-CGCTTCACGAATTTGCGTGTCAT3′; FAM64A forward, 5′-CCTGGAAACGCCTGGAAAC-3′ and reverse 5′-CAAAGCACTCTTAGCTGAGCG-3′. The 2^−ΔΔCq^ method was used to determine the expression level of miR-493 and FAM64A.

### 2.10. Western Blot Analysis

Cellular lysates were electrophoresed in a 10% SDS-PAGE gel and then transferred to nitrocellulose membrane (GE Healthcare). Primary antibody anti-CATS 2C4 (1 : 1000) and GAPDH were purchased from Abcam. The membranes were rinsed with TBS (Tris-Buffered Saline) and Tween (Sigma-Aldrich) twice before being incubated with Goat Antimouse IgG H&L (HRP) for 1 h at room temperature in the dark. A Bio-Rad ChemiDoc™ XRS system was then used for membrane visualization.

### 2.11. Wound Healing Assay

The cells with a density of 8 × 10^4^ cells/well were seeded in a 24‐well plate. A vertical line was drawn among them with a sterile pipette tip after approximately 80% of the confluency was reached. The suspended single cells on the surface were then washed away with warm PBS. Fresh DMEM containing 10% FBS was added to plates, and the cells were then cultured in an incubator at 37°C with 5% CO_2_. The cells were imaged under a phase contrast light microscope, at 0, 24, and 48 h, respectively. Cells' migration ability was then measured with Image J (National Institutes of Health).

### 2.12. Statistical Analysis

All data in this study were expressed as mean ± SD (standard deviation). SPSS 22.0 was used throughout this study for the statistical analysis, and one‐way analysis of variance was used for comparison. Survival of mice in this study was measured with Kaplan–Meier analysis. A *P* < 0.05 was designated as statistically significant.

## 3. Results

### 3.1. Overexpression of FAM64A in OS Tissues Predicted Poor Prognosis

**Figure 1:**
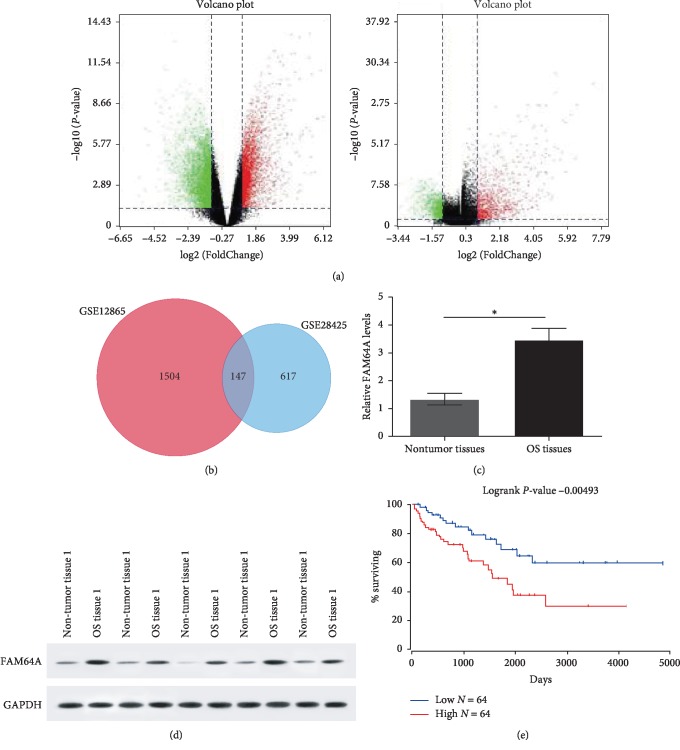
Expression of FAM64A in tumor tissues. (a) Differentially expressed genes were selected on GEO. (b) A total of 147 genes were obtained. (c) Gene expression of FAM64A in OS tissues and nontumor tissues using RT-RCR. (d) Protein expression of FAM64A in 5 pairs subject using western blot. (e) Kaplan plot for FAM64A in SARC. ^*∗*^*P* < 0.05. OS, osteosarcoma.

As shown in Figure 1(a), GSE12865 and GSE28425 were selected on GEO to select differentially expressed genes, with *P* < 0.05 and logFC absolute value >1.5. There were 1,504 GSE28425 differentially screened genes GSE12865 and 617 intersections, and a total of 147 genes were obtained (Figure 1(b)). The expression level of FAM64A in OS tissues and adjacent nontumor tissues were analyzed. As demonstrated in Figures 1(c) and 1(d), the gene expression level of FAM64A and its protein in tumor tissues were significantly heightened compared with the control sample (*P* < 0.05). To GO annotate these 147 genes, we focused on this FAM64A. GO: 0009987 cellular processes. Online software (http://www.oncolnc.org) was used to analyze the SARC data of TCGA, and it was found that the difference of KM curve logrank analysis between patients with high and low expression of this gene, suggesting patients with higher expression of FAM64A, had poor outcome (Figure 1(e)).

### 3.2. Overexpression of FAM64A Promoted Proliferation, Migration, and Invasion of OS Cells

**Figure 2:**
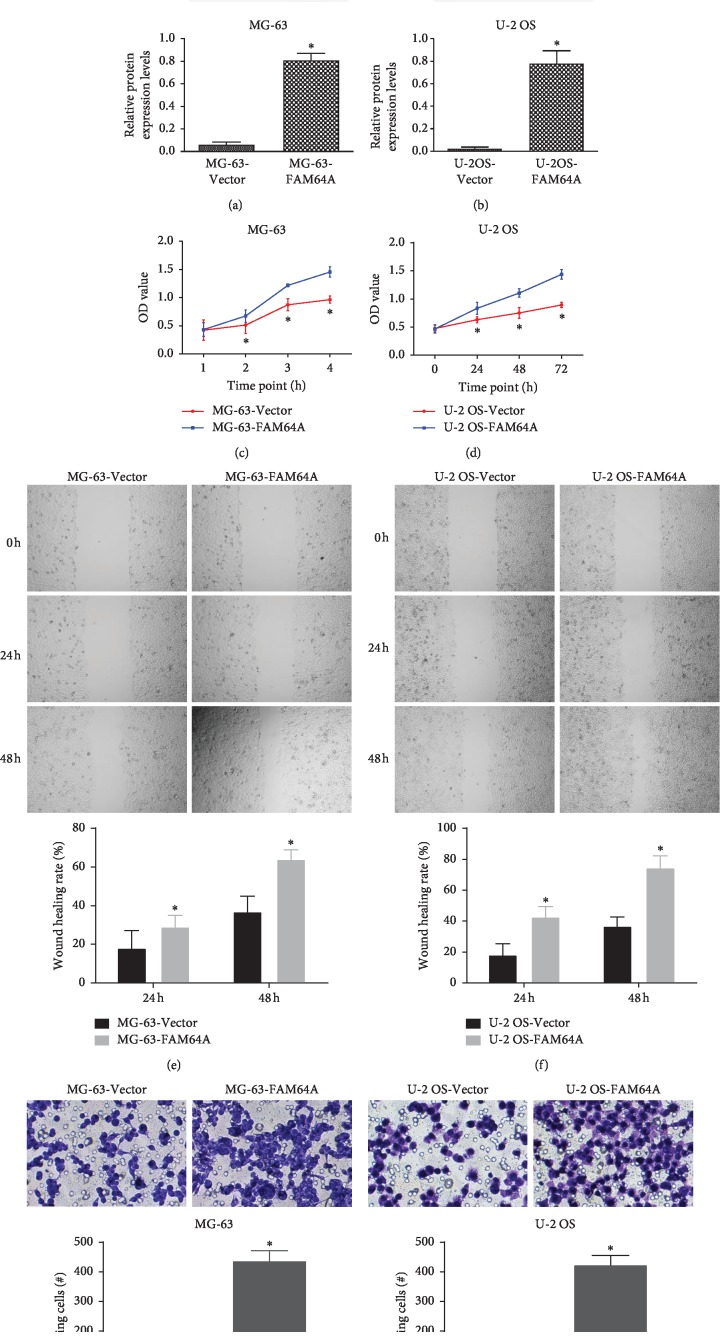
Upregulation of FAM64A promoted the tumorigenicity of OS cells. (a) and (b) Protein expression of FAM64A was detected after vector and FAM64A plasmid transfection. (c) and (d) Proliferation of tumor cells was determined by CCK-8 assay. (e) and (f) Migration was measured by the wound healing assay. (g) and (h) Invasion of cells was analyzed using a Transwell assay. ^*∗*^*P* < 0.05.

To further investigate the role of FAM64A in OS, OS cells were transfected with plasmid. The data of western blot confirmed that FAM64A was significantly upregulated in MG-63 and U-2 OS cells (Figures 2(a) and 2(b), *P* < 0.05). The CCK-8 assay and Transwell invasion assay were performed to verify the influence of overexpression of FAM64A on OS cell proliferation and invasion. As shown in Figures 2(c) and 2(d), MG-63 and U-2 OS cells transfected with FAM64A had a significantly higher proliferation rate than cells transfected with vector (*P* < 0.05). Migration assay demonstrated that the migrated numbers of MG-63 and U2OS cells transfected with FAM64A were significantly higher compared with the cells transfected with vector (Figures 2(e) and 2(f); *P* < 0.05). Additionally, invasion assay indicated that invasive abilities were markedly heightened in MG-63 and U2OS cells transfected with FAM64A plasmid (Figures 2(g) and 2(h); *P* < 0.05).

### 3.3. Silencing FAM64A Inhibited Proliferation, Migration, and Invasion of OS

**Figure 3:**
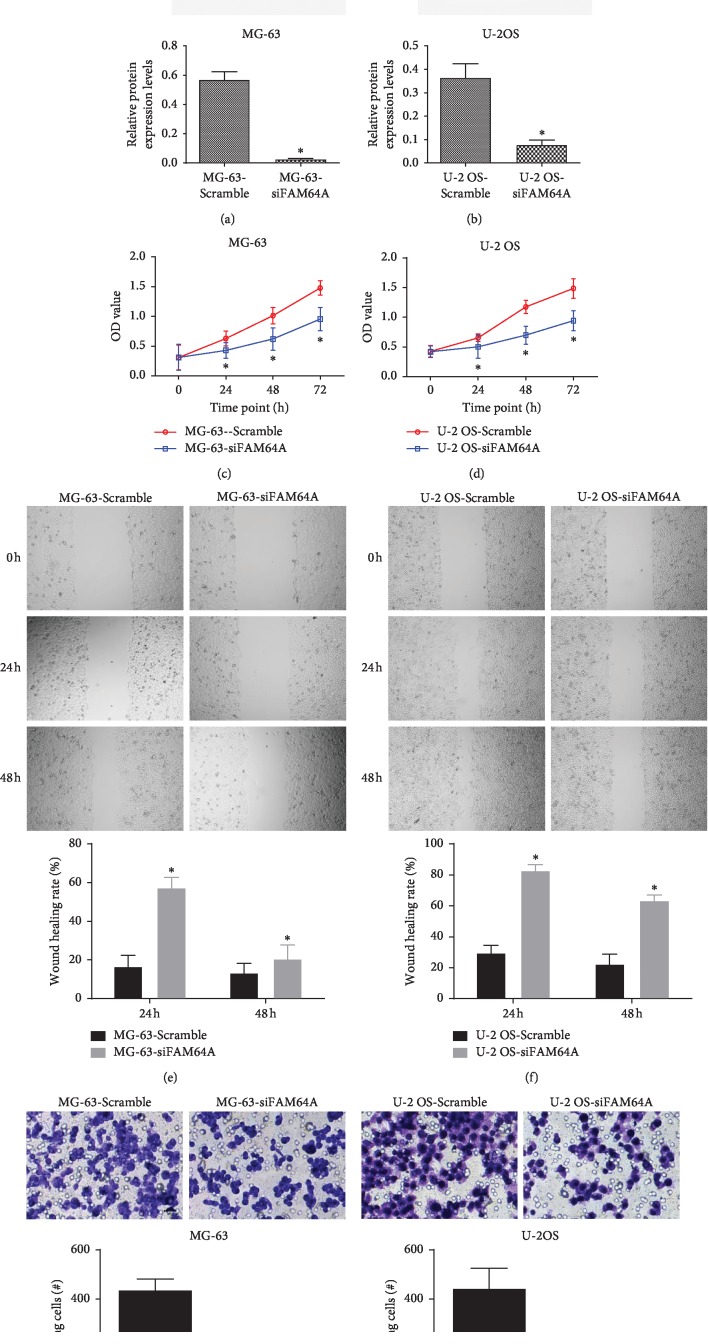
Functional effects of FAM64A siRNA knockdown on MG-63 and U-2 OS cells. (a) Protein expressions of FAM64A in si-control and si-FAM64A transfected cells. (g) and (h) migration assay of si-FAM64A transfectants. Representative photomicrographs are shown at 100 magnification.

To verify the mode of action of FAM64A, siRNA was utilized to downregulate its expression in mg-63 and U-2 OS cells (Figures 3(a) and 3(b), *P* < 0.05). The introduction of FAM64A siRNA into MG-63 and U-2 OS cells resulted in impeded tumor cell proliferation (Figures 3(c) and 3(d); *P* < 0.05), migration (Figures 3(e) and 3(f); *P* < 0.05), and invasion (Figures 3(g) and 3(h); *P* < 0.05), compared with the Scramble group.

### 3.4. Silencing FAM64A in Mice Inhibited OS Tumor Growth

**Figure 4:**
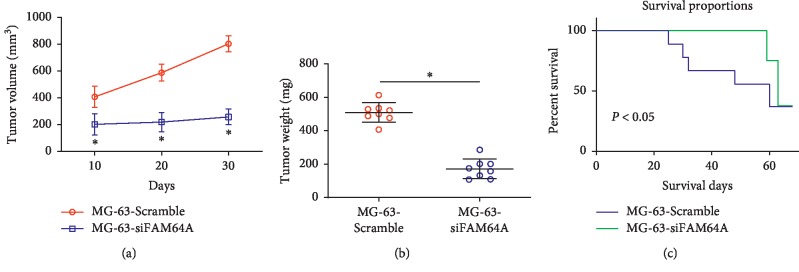
Silencing FAM64A in mice. (a) Tumor volume of mice burden MG-63-Scramble/MG-63-siFAM64A cells was measured at 10, 20, and 30 days. (b) Mice were sacrificed after 30 days, and tumor weight of mice burden MG-63-Scramble/MG-63-siFAM64A cells was analyzed. (c) Survival rate of mice. ^*∗*^*P* < 0.05.

We explored the regulatory effects of FAM64A in vivo. As displayed in Figures 4(a) and 4(b), si-FAM64A treatment significantly decreased the volume of tumor and its weight compared with the Scramble group (*P* < 0.05). The Kaplan–Meier survivor function revealed that Silencing FAM64A significantly improved the survival of tumor-bearing mice (Figure 4(c), *P* < 0.05).

### 3.5. FAM64A Is the Target of miR-493

**Figure 5:**
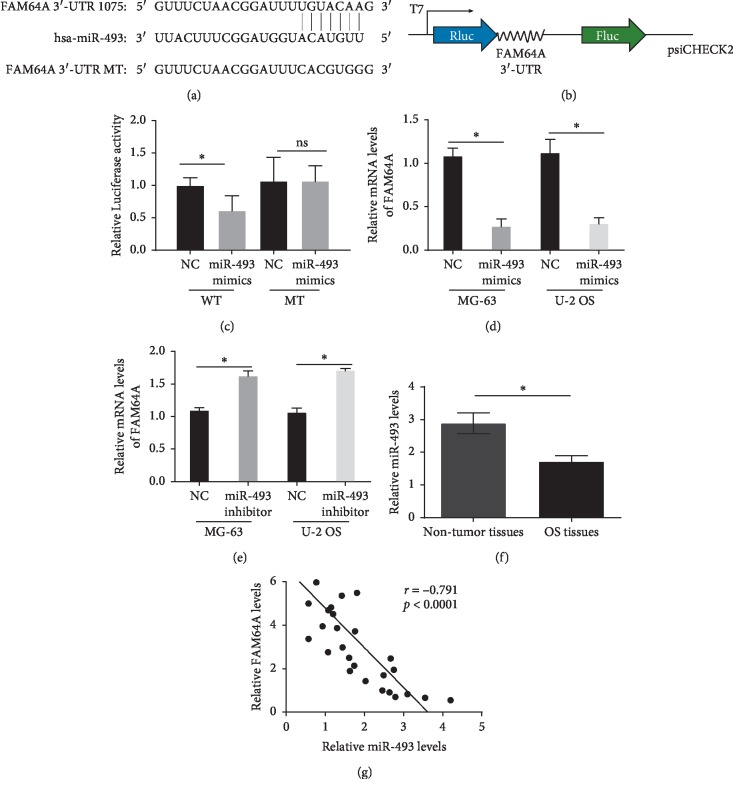
FAM64A is the target of miR-493 in OS. (a) MiR493 binding sequence in 3′UTR of the FAM64A gene and the mutant sequence. (b) The pattern of FAM64A 3-UTR WT/MT inserted to psiCHECK2. (c) 293T cells were cotransfected with psiCHECK2-FAM64A 3′-UTR WT or psiCHECK2-FAM64A 3′-UTR MT and miR493 mimics or NC. The luciferase activity was measured. (d) and (e) mRNA levels of FAM64A in MG-63 and U-2 OS cells. (f) Detection of miR-493 expressions in OS tissues and nontumor tissues using RT-RCR; (f) correlation analysis of miR-493 levels with FAM64A mRNA levels. ^*∗*^*P* < 0.05. UTR: untranslated region; WT: wild type; MT: mutant; NC: negative control.

Through bioinformatics analysis, we found that many miRNAs may regulate FAM64A. MiR-493 has been proved to play the role of tumor suppressor gene in osteosarcoma and inhibit the cell biological process of osteosarcoma, playing an opposite role with FAM64A [8, 9]. So, we chose miR-493 to study its regulation on FAM64A expression. Our data showed that contains a potential complimentary binding site for miR-493 within FAM64A 3'-UTR (Figure 5(a)). The possible participation of miR-493 in the FAM64A pathway is indicated in Figure 5(b). The data of luciferase activity showed that luciferase activities were significantly decreased in 293T cells after transfection with psiCHECK2-FAM64A 3'-UTR WT and miR-493 mimics (Figure 5(c), *P* < 0.05). We also analyzed the levels of FAM64A in MG-63 and U-2 OS cells transfected with miR-493 mimics, inhibitor or NC. 48 h after transfection, gene expression at mRNA levels were measured with real-time PCR. As demonstrated in Figures 5(d) and 5(e), FAM64A mRNA levels were significantly alleviated in mimics, while FAM64A mRNA levels were increased when burden inhibitor (both *P* < 0.05). The effect of mimics was suppressed by MT, suggesting miR-493 targeting to FAM64A 3'-UTR. We also showed that miR-493 expressions were downregulated in OS tissues and negatively correlated with FAM64A mRNA expressions (Figures 5(f) and 5(g), *P* < 0.05).

### 3.6. MiR-493 Inhibits Proliferation, Migration, and Invasion of OS Cells via Regulating FAM64A

**Figure 6:**
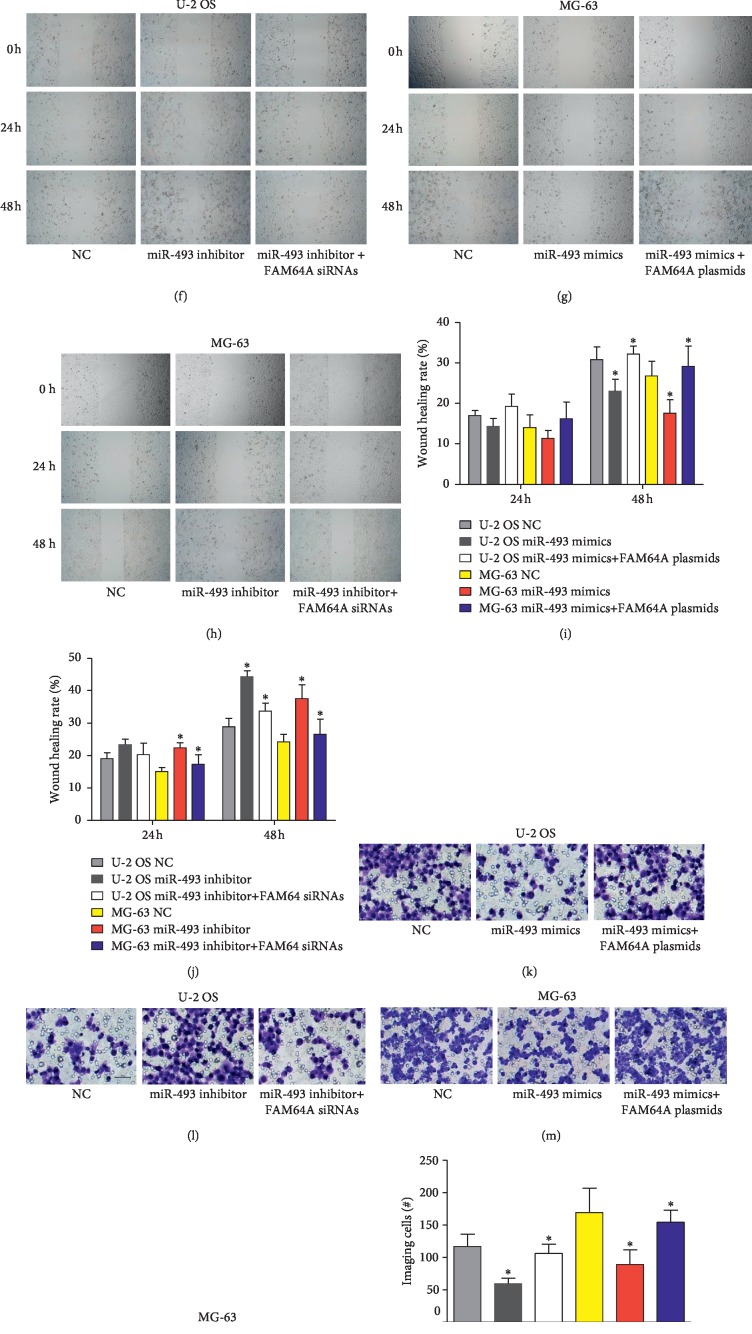
miR-493 inhibits proliferation, migration, and invasion of OS cells via regulating FAM64A. (a–p) U-2 OS and MG-63 cells were transfected with miR-493 mimics and miR-493 inhibitors with FAM64A plasmids and FAM64A siRNAs. (a–d) Proliferation of tumor cells was determined by CCK-8 assay. (e–j) Migration was measured by wound healing assay. (k–p) Invasion of cells were analyzed using a Transwell assay. ^*∗*^*P* < 0.05.

To investigate the role of miR-493/FAM64A in OS, OS cells were transfected with miR-493 mimics and miR-493 inhibitors with FAM64A plasmids and FAM64A siRNAs. The CCK-8 assay, wound healing assay and Transwell invasion assay were performed to investigate the proliferation, migration, and invasion. The results suggested that miR-493 significantly influenced the proliferation (Figures 6(a)–6(d)), migration (Figures 6(e)–6(j)), and invasion (Figures 6(k)–6(p)) of MG-63 and U-2 OS cells, while FAM64A plasmids and FAM64A siRNAs could attenuate the effects of miR-493 mimics and miR-493 inhibitors, respectively (Figures 6(a)–6(p)). These assays demonstrated that miR-493 inhibited proliferation, migration, and invasion of OS cells via regulating FAM64A.

## 4. Discussion

Recent studies have shown that FAM64A, also named as CATS and PIMREG, participates in malignant transformation [10–13]. FAM64A was first studied in hematologic carcinomas, which was known as CALM/AF10 interacting proteins. As reported, higher levels of FAM64A was associated with the tumor proliferation process. However, the role of FAM64A in solid tumor is still few. Here, we evaluated the gene expression profiling of OS in the database and found the FAM64A. Based on the data from GEO, we chose FAM64A and evaluated its expression in tumor samples. We found elevated expression of FAM64A in tumor tissues in comparison to the nontumor tissues.

Moreover, we identified the promotion of tumorigenicity by establishing overexpressing FAM64A OS cells model, which indicated underlying molecular mechanism of how miR-493 participated in upregulating migration and invasion of tumor cells. Previous evidence suggested that miR-493 inhibits the biological behavior of lung tumor [14]. Meanwhile, miR-493 also acts as a suppressor in various cancers including colon cancer, bladder cancer, and ovarian cancer via multiple intracellular signaling pathways [15–17].

Next, we performed a xenograft model of tumor and further investigated the prognostic role of FAM64A in mice. We found a sharp decrease of tumor volume and tumor weight in siRNA-transfected mice, indicating silencing FAM64A suppressed tumor growth. Kaplan–Meier curves for OS *in vivo* revealed that upregulation of FAM64A was positively correlated with worse outcomes. To confirm the interaction of FAM64A and miR-439, we performed the luciferase reporter assay. The effects of FAM64A knockdown on OS cells mimicked those induced by miR-493 mimics and were reversed by miR-493 inhibitor.

Taken together, our data demonstrated that miR-493 negatively regulates FAM64A via binding to its 3′ UTR in OS. Our study found that higher expression of FAM64A *in vitro* and *in vivo* is correlated with poor survival in OS. This is the first study to shed light on the participation of FAM64A regulated by miR-439 in the malignancy of osteosarcoma.

## Data Availability

The data and materials used to support the findings of this study are included within the published article.
